# Comment on de la Monte et al. Betel Quid Health Risks of Insulin Resistance Diseases in Poor Young South Asian Native and Immigrant Populations. *Int. J. Environ. Res. Public Health* 2020, *17*, 6690

**DOI:** 10.3390/ijerph18168750

**Published:** 2021-08-19

**Authors:** Nansi López-Valverde, Antonio López-Valverde

**Affiliations:** Department of Surgery, University of Salamanca, Instituto de Investigación Biomédica de Salamanca (IBSAL), 37007 Salamanca, Spain; nlovalher@usal.es

In September 2020, de la Monte and colleagues published the article “Betel Quid Health Risks of Insulin Resistance Diseases in Poor Young South Asian Native and Immigrant Populations”, doi:10.3390/ijerph17186690, in the *International Journal of Environmental Research and Public Health*. The study reviewed, in Southeast Asian populations, the increase in chronic consumption of betel quid, along with tobacco, as a source of increased insulin-resistant pathologies [1].

We have carefully consulted the text of the article and consider that certain bibliographic citations are associated with paragraphs that have nothing to do with the text in which they are cited.

In order not to be tedious, we will refer to three demonstrative and disturbing examples:

(A). De la Monte and colleagues state at the end of Section 4. Betel Quid Preparations: *“Although all age groups were affected, the greatest impact of increased areca nut availability/production was in youths from socially and economically disadvantaged populations”,* referenced with citation 12. We have carefully read the article “Global epidemiology of areca nut usage” [2] and at no point do the authors refer to the increased availability/production of areca nuts in young people from socially and economically disadvantaged populations. They only refer in the Global Epidemiology section to the wide range of point prevalence of areca chewing and the demographic differences observed; they conclude by stating that consumption is increasing in countries such as Taiwan or India, which increases the risk of oral cancer.
(B). At the end of Section 5. Cultural and Medicinal Uses of Areca Nut and Betel Quid: *“However, of particular interest are the claims that betel quid enhances memory and cognition”,* referenced with citation 19. Similarly, we consulted the article “*Areca catechu* L. (Arecaceae): a review of its traditional uses, botany, phytochemistry, pharmacology, and toxicology” [3] and found no specific reference to the effects of the alkaloid on memory or cognition, as stated by de la Monte and colleagues.
(C). Finally, in Section 9. Adverse Health Effects of Chronic Betel Quid and Tobacco Exposures, they say: *“betel quid chewing during pregnancy significantly impaired fetal development, increased risk of low birth weight, and led to neonatal withdrawal syndrome”,* reference 91, “Consumption of the tobacco–betel–calcium hydroxide mixture, among Spanish students” [4]. This scientific letter was published by us in 2018 as a preliminary study on the consumption of the tobacco–betel–calcium hydroxide mixture among Spanish students, based on five surveys carried out in strategic cities in Spain. In the study, we highlighted the worrying new forms of tobacco consumption in Spain among the youth population, mainly imported from other countries, due to multiculturalism and migration to our country, without referring at any time to the potential harm to the foetus, or the increased risk of low birth weight or neonatal abstinence due to the consumption of betel quid during pregnancy.

Bibliographic references and citations within scientific papers and documents (especially review articles) tell potential readers where the authors have taken their information from. A good scientific paper is one that is well documented; it also shows that the authors have taken care to read the referenced documents, thus providing reliability to the study, but if the references are cited out of context in order to bias them in favor of one’s own argumentation, there is a risk that readers who know the subject matter will consider that they are facing situations of misinterpretation of the sources, with the consequent loss of confidence in the article in question.

In view of the above, we are of the opinion that the results of the de la Monte and colleagues’ study, should be taken with caution.
